# Antibiotic Residues in UK Foods: Exploring the Exposure Pathways and Associated Health Risks

**DOI:** 10.3390/toxics12030174

**Published:** 2024-02-24

**Authors:** Jegak Seo, Frank Kloprogge, Andrew M. Smith, Kersti Karu, Lena Ciric

**Affiliations:** 1 Healthy Infrastructure Research Group, Department of Civil, Environmental and Geomatic Engineering, University College London, Gower Street, London WC1E 6BT, UK; 2 Institute for Global Health, University College London, Rowland Hill Street, London NW3 2PF, UK; 3 Eastman Dental Institute, University College London, Rowland Hill Street, London NW3 2PF, UK; 4 Department of Chemistry, University College London, 20 Gordon Street, London WC1H 0AJ, UK

**Keywords:** diet survey, food contamination, antibiotic residues, low-temperature partitioning extraction, exposure modelling

## Abstract

While the use of antibiotics has been reported as extensive in the rearing of agricultural animals, insufficient information is available on the antibiotic residues in animal products and the adverse impact that consistent low-level exposure to antibiotics might have on the human body and its microbiome. The aim of this study was to estimate the antibiotic concentrations that humans are exposed to via their diet using the concentration of antibiotics in animal food products and water and an online survey on dietary intake. A total of 131 participants completed the dietary intake survey, with the majority belonging to the omnivorous diet group (76.3%). Distinct dietary trends were observed in the omnivorous and unknown groups eating animal products, with specific food types dominating each meal: pork (e.g., ham) and dairy products (e.g., milk, yoghurt) during breakfast, beef (e.g., burgers) and chicken (e.g., chicken breast) products during lunch, and fish (e.g., salmon fillet) during dinner. In total, 34 different animal-based food and drink products were tested for the presence of ten different antibiotics. Of all the products tested, over 35% exceeded the acceptable daily antibiotic intake for amoxicillin, ampicillin, and enrofloxacin.

## 1. Introduction

The widespread use of antibiotics in animal husbandry, driven by growth promoters and disease treatments, has raised significant concerns over the exposure risk associated with the consumption of animal products [1,2,3]. One of the main concerns about the extensive use of antibiotics is antimicrobial resistance (AMR), which poses a significant global threat and has the potential to become the next pandemic [4]. Currently, AMR is responsible for 700,000 deaths annually, and this number is projected to escalate to 10 million deaths per year by 2050, as highlighted in a report from the government of the United Kingdom by Jim O’Neil in 2016 [5]. Extensive research efforts have primarily concentrated on the direct intake of antibiotics by humans through prescriptions, pharmacy purchases, and hospital use [6,7,8]. Importantly, excessive agricultural and veterinary antibiotic usage contributes to the pervasive presence of veterinary antibiotic residues in animal products globally [9,10,11,12]. This trend not only reflects the growing concern but also perpetuates the vicious cycle of increasing veterinary antibiotic use, which accelerates the development of antibiotic resistance in animals [10]. Challenges in controlling and regulating the purchase and use of veterinary antibiotics in many countries further exacerbate this situation [1], leading to inappropriate administration of antibiotics without compliance with prescribed withdrawal periods [13]. These practices underscore the urgent need for comprehensive strategies to mitigate the risks associated with antibiotic residues in the food chain [14].

Antibiotics have been detected in food products and drinking water due to the wide range of antibiotics used not only in the treatment of infectious diseases but also in agricultural run-off, wastewater treatment, nonmedical applications, and open defecation [1]. The identification and quantification of antibiotics in food and drinking water has become a new field of study, exploring the undiscovered world which has been harmfully polluted by humans with a variety of antibiotics since the 2000s [15]. A recent comprehensive review of antibiotic monitoring studies conducted throughout the world identified residues of antibiotics which are used in humans and animals, meat and dairy products, and plants and drinking water [1,16]. Antibiotics administered to humans are frequently detected in food and drinking water, and their presence is also often observed in plants, likely due to exposure through irrigation or the use of fertilizers derived from wastewater and manure [17,18]. These studies suggest that the risk of AMR through the chronic consumption of a trace level of antibiotics in foods or drinks is significant.

The overall aim of this study was to estimate the daily intake of antibiotic residues via diet intakes, using the antibiotic concentrations present in drinking water and animal-based food products from the UK, to establish a measure of the subsequent risk of human exposure. Specifically, this research aimed to explore the antibiotic concentration in food products, including beef, pork, chicken, fish, dairy products, and drinking water, by monitoring questionnaires and performing an analysis of food samples collected from local stores. Therefore, this study was delivered in terms of three specific objectives: (1) to characterise, using an online questionnaire, how the UK public population comes into contact with antibiotics through their consumption of animal products and drinking water; (2) to determine the levels of antibiotic concentrations in animal products and drinking water by collecting samples from large supermarket chains; and (3) to analyse the range of antibiotic exposure in survey participants by combining the data on their dietary intakes with the concentration of antibiotic residues in animal products and drinking water.

## 2. Materials and Methods

### 2.1. Online Diet Survey

An online survey was used as it is a direct method for dietary assessment which collects primary dietary data from individuals [19]. University College London (UCL) Opinio (https://opinio.ucl.ac.uk/admin/folder.do (accessed on 10 March 2021)) was used to apply a quantitative method to determine both the types and amounts of food consumed. All data collections were performed in accordance with the relevant guidelines and regulations. Informed consent was secured from every participant before they accessed the questionnaire.

Two days of 24 h recall and a food frequency questionnaire (FFQ) were the main survey channel, with a retrospective approach; an estimated food record and weighed food record were included as subsidiary functions using Likert scales and open-ended questions [20]; and innovative technologies supported by any devices were used to support the technical approaches of the participants and increase the accuracy of the survey. In the 24 h recall section, participants were asked to recall their dietary intake for 48 h in total. Twenty slots for food and drinks, per day, were provided to ensure sufficient opportunities to record all animal-based food and drinks consumed. Time, place, type and name of product, and volume (ml) or mass (g) of product were required for each different food or drink type. After 24 h recall, the FFQ was assessed to investigate the frequency with which foods and drinks, and/or food groups were consumed over a certain time period. After completing the two sections, the participants were asked to compare their dietary history to their general intake in a week using Likert scales. Firstly, the participants were asked to estimate the number of intake days per week. Then, the amount of each recorded food and drink was compared to the general intake in a week by rating in percentage, on a Likert scale of between less than 10% and more than 200%.

In accordance with the FAO guidelines (2018), the survey was designed to facilitate a nuanced analysis of results [19]. The focus was placed on the detailed collection of dietary histories, pivotal for the estimation of antibiotic consumption. The US FDA’s Estimated Meal Intake formula (Equation (1)), which standardizes the weight assumption for an adult participant at 60 kg, was utilized for this purpose [21]. In this research, the reference to 12 o’clock was intended to encompass the time range between 1200 and 1259, and, similarly, other hourly references were aligned with their respective one-hour time intervals.

### 2.2. Antibiotic Quantification in Food and Drink

#### 2.2.1. Antibiotics, Chemicals, and Reagents

The following antibiotics (CAS number): tetracycline (64-75-5; TC), oxytetracycline (6153-64-6; OTC), amoxicillin (61336-70-7; AMOX), ampicillin (7177-48-2; AMP), trimethoprim (738-70-5; TMP), sulfadiazine (68-35-9; SDZ), ciprofloxacin (85721-33-1; CIP), enrofloxacin (93106-60-6; ENR), erythromycin (114-07-8; ERY), and tylosin (1405-54-5; TYL) were purchased from Sigma-Aldrich (St. Louis, MO, USA), all with purity ≥ 99%. All chromatographic-grade reagents, including acetonitrile (ACN), trifluoroacetic acid (TFA), water, and formic acid (FA), were used for LC–MS analysis with purity higher than 99.8% and were purchased from Fisher Scientific (Lancashire, UK).

#### 2.2.2. Low-Temperature Partitioning Extraction (LTPE)

All samples were purchased from large supermarket chains in London, United Kingdom. The samples were purchased at the same time as when the survey was opened to participants, i.e., from 28 May 2021 to 30 July 2021 and from 12 January 2022 to 17 March 2022. The LTPE method was performed to extract potential antibiotic residues from samples in a cost-effective manner. Additionally, the cold environment during the extraction process minimizes the thermal degradation of target compounds [22].

For all sample preparation, at least 3.0 g of a whole food sample was homogenized using a kitchen blender (BOSCH, MSM6B150GB) for 1 min in triplicate. For the drink sample, an unopened bottle of the drink product was inverted upside-down 20 times before aliquoting into replicates. Briefly, 1.0 g of the homogenized sample was aliquoted to a 50 mL test tube. Aliquoted replicates were further homogenized using pellet pestles (Bel-ART SP SCIENCEWARE, 19923-000) for 1 min. The processed sample vials were covered with aluminium foil and stored at −20 °C prior to analysis. Then, 1.0 g of HPLC-LiChropur™ NaCl (Merck, 7647-14-5) was added to the tube and vortexed at 448× *g* for 1 min, followed by the addition of 8.0 mL of 50% ACN, 47.5% water, 2.5% TFA (Honeywell, 19182-250 mL) to the tube. The tube was vortexed and centrifuged for another 5 mins. The prepared samples were stored at −20 °C freezer overnight. Then, 1.5 mL of the organic phase was removed and transferred to a 2.0 mL microcentrifuge tube. The samples were centrifuged at 3278× *g* for 10 min at 25 °C, and 1.0 mL of the supernatant was transferred to individual HPLC glass vials for LC–MS analysis.

#### 2.2.3. LC–MS Analysis

The samples were analysed using a liquid chromatography tandem mass spectrometry (LC–MS/MS) instrument. The instrument consisted of an Accela LC system connected to a Finnigan Linear Trap Quadrupole (LTQ) Linear Ion Trap mass spectrometer from Thermo Fisher Scientific, UK [23]. The chromatographic separation was achieved using a Hypersil GOLD C18 column (150 mm × 2.1 mm, 1.9 μm; Thermo Fisher Scientific, UK). The column temperature was maintained at 30 °C. Mobile phases A and B were the following: (A) water with 0.1% FA and (B) ACN with 0.1% FA, and the flow rate was 200 μL/min. The gradient program was as follows: 2% of B for the first 2 mins and a gradual change to 98% B in 16 mins, then changed to 2% of B in 0.1 mins and remained at 2% B for another 1.9 mins. The total run time was 20 mins per sample. The injected sample volume was 10 μL. The liquid effluent from the C18 column was directed into the electrospray (ESI) source of the LTQ mass spectrometer (MS). The ESI was in positive mode, and the source parameters were as follows: a spray voltage of 4500 V, capillary temperature set to 280 °C, sheath gas at a pressure of 40 psi, ion sweep gas pressure (0 psi), >99% purity of N_2_ auxiliary gas set at 5 psi, and a skimmer offset at 25 V. The data were collected using a full-scan MS event with a mass range from *m*/*z* 50 to 1000 and in the MS/MS event, which was setup for each *m*/*z* value corresponding to each antibiotic, as per Figure S1. The isolation width (*m*/*z*) was 2.0 and the collision energy was 35%. The analytical batch was set up containing water blanks (H_2_O), which were analysed after each sample analysis, and a quality control consisting of a pure antibiotic at a concentration of 10 µg/L.

#### 2.2.4. Method Validation

Figure S1 shows the chromatographic separation of 10 antibiotics on the C18 column, and their retention times are summarized in Table S2. The LC–MS method validation parameters, such as accuracy, limit of detection (LOD), and limit of quantification (LOQ), were calculated and are summarized in Supplementary Materials Table S1. For calibration curves, five replicates at nominal concentrations of 50, 100, and 500 μg/L were prepared and analysed by LC–MS. The accuracy (%) and relative standard deviation (RSD; %) of the measurements were determined, and the calibration curves were constructed for each antibiotic (Table S2). The accuracy and RSD ranged from 97.2 to 111.22% and 0.01 to 0.92%, respectively.

The LTPE validation is summarized in the Supplementary Materials. Pork chop meat was used as a pure matrix to compare the accuracy of the LTPE methods. Triplicates of the non-spiked pure matrix were tested using LTPE methods to determine the presence of antibiotics. Triplicates of the pure matrix were spiked at nominal concentrations of 100 μg/L of 10 antibiotic mixture solution, and the antibiotics were extracted using the LTPE method. Linear regression analysis was performed to calculate the linearity (R^2^ > 0.999) of the calibration curves using Microsoft Excel version 16.53 (Microsoft Excel, 2021), and the results are summarized in Table S3. The recovery of the LTPE method using 100 ug/kg of a 10 antibiotic mixture stock solution was between 87.6 and 93.5%, and the recovery using triplicates of pork chop matrix spiked with a 100 μg/kg of a 10 antibiotic mixture was between 89.6 and 95.4% (Table S3). Method validation procedures, including recovery experiments at the LOQ, were confirmed to be in full compliance with the residue analysis guidelines as outlined in the European Commission document, ensuring satisfactory performance for detecting antibiotic residues from animal origin samples [24].

### 2.3. Estimated Meal Intake (EMI)

The estimated daily intake formula from the US FDA [21] was modified to calculate the antibiotic intake from each meal using Equation (1) instead of the total intake of substances in a day. Also, additional dilution factors such as the average volume of drinks and meals, stomach acid, and bile juice in the human digestive system were taken account of to determine the luminal concentration of antibiotics in the human duodenum. This is an example of an equation:

(1) $${\mathrm{EMI}}_{x}=\sum_{f=1}^{F}(\frac{{freq}_{f}\times {Port}_{f}\times {Conc}_{xf}}{N}\times \frac{{Port}_{f}}{V_{total}})$$

where the total number of foods in which antibiotic “*x*” can be found is expressed as *F*. *Freq_f_* represents the average portion size for food “*f*”. *Port_f_* shows the number of occasions when food “*f*” was eaten over “*N*” meals during the survey. The concentration of antibiotic “*x*” in food “*f*” is denoted as *Conc_xf_*. *N* expresses the total number of meals in the survey. Then, *V_total_* represents the sum of the average volume of drinks, the average volume of a meal, and the average volume of human stomach juice (60 mL).

The most frequently consumed meat type was chosen as the representative food type for each meal. The list of consumed foods and drinks was determined for each meal over the 48 h diet survey. The detected antibiotics were determined from the specified foods and drinks, and the average concentrations of the detected antibiotics were applied. However, any concentrations below the acceptable daily intake (ADI) concentration (https://apps.who.int/food-additives-contaminants-jecfa-database/ (accessed on 6 April 2022)) were excluded from the list for each food item. The total volume of each meal was calculated by adding the average volume of the consumed drinks and foods during a meal and the average volume of gut juice, 60 mL, which is the volume when the human stomach is empty [25].

### 2.4. Statistical Analysis

Regression analysis was performed to determine the accuracy and validity (R^2^ > 0.999) of calibration curves for antibiotic measurement using LC–MS. The mean differences in food and drink consumption on different days and in seasons were statistically analysed and compared by a one-way ANOVA test with a post hoc Bonferroni test.

## 3. Results

### 3.1. Demographical Profiles and Overall Consumption Trend

The online survey investigating participants’ dietary intakes was conducted over 48 h periods during two seasons: from 28/05/2021 to 30/07/2021 (summer, *n* = 51) and from 12 January 2022 to 17 March 2022 (winter, *n* = 80), as detailed in Table 1. All participants (*n* = 131) agreed to the UCL General Research Participant Privacy Notice.

In this research, the total participant count was adjusted to 117 (45 participants from the summer survey and 72 participants from the winter survey), encompassing both omnivores and individuals with unknown dietary intakes, as it provided a diverse representation of animal product dietary intakes and a statistically sufficient number of respondents [19,26].

### 3.2. Meat Consumption

**Figure 1:**
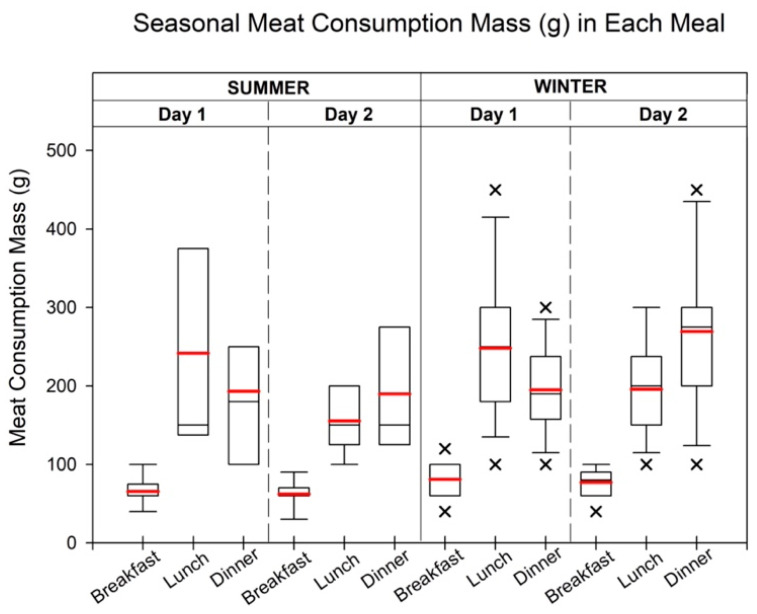
The meat intake volume (g) in each meal during summer and winter. Each meat intake time was selected by the highest overall meat consumption in each meal during the summer and winter. The box ranges from the 25th to 75th percentiles, and the ends of whiskers represent minimum and maximum data values, excluding outliers. Black and red lines show the median and mean values of intake, respectively. Cross (×) indicates the outliers which are more than 1.5 times of the interquartile range. During the summer mornings, pork consumption varied between 60 and 90 g, averaging around 88 g. For lunch on the first day, chicken and beef showed comparable totals, each with a minimum of 100 g. Chicken’s maximum consumption, however, was notably higher, at 600 g. Fish dominated dinner on the first day with a maximum of 400 g, whereas the mean fish consumption matched the maximum pork intake. On the second day, chicken consumption (400 g) surpassed pork consumption (300 g), with similar mean values. Winter consumption mirrored these patterns, with pork exclusive to breakfast (60 g minimum), with a higher average on the first day (81.3 g), and chicken and beef prevailing in lunch (minimum 100 g).

Based on the overall meat consumption trend, the peaks of meat consumptions are shown in Figure 1. In summer and winter, both had the same pattern of food product type in each meal, for instance, pork, chicken, and fish for day 1 and pork, beef, and chicken for day 2. Detailed meat product consumption information is provided in Table S4.

During the summer, breakfast consumption of pork showed variations with minimum quantities of 60 g and maximum quantities of 90 g and 80 g, respectively. The average breakfast consumption for each day was approximately 88 g. At lunch on the first day, chicken and beef had comparable total consumption, with minimum values of 100 g for both types. However, chicken had a significantly higher (*p* < 0.05) maximum consumption, of 600 g, than beef. The average consumption of chicken (250 g) was slightly higher than that of beef (200 g). For lunch on the second day, beef consumption at 12 o’clock was lower than the consumption at 13 o’clock, although the maximum, mean, and median values (250 g, 190.5 g, and 200 g) were higher during the earlier time period. In terms of dinner on the first day, fish was the most consumed meat type, with a maximum quantity of 400 g. The mean consumption of fish (200 g) matched the maximum pork consumption. On the second day, chicken consumption (400 g) exceeded pork consumption (300 g), with similar mean and median values for both.

Similar consumption patterns were observed during the winter, with pork being the only meat consumed during breakfast. The minimum pork consumption remained the same at 60 g for both days, but the average consumption was slightly higher on the first day (81.3 g) than on the second day (77.1 g). For lunch on each day, chicken and beef were the predominant meat types consumed. On the first day, chicken consumption exceeded fish consumption by 108%, whereas on the second day, beef consumption was more than four times higher than other meat types. The mean lunch consumption for chicken and beef was 248.1 g and 195.8 g, respectively. During dinner on the first day, fish consumption was more than double that of the second most consumed meat, chicken. However, on the second day, beef consumption was significantly higher (*p* < 0.05). The mean consumption of fish and chicken during dinner was 195.0 g and 269.2 g, respectively.

### 3.3. Dairy Consumption

**Figure 2:**
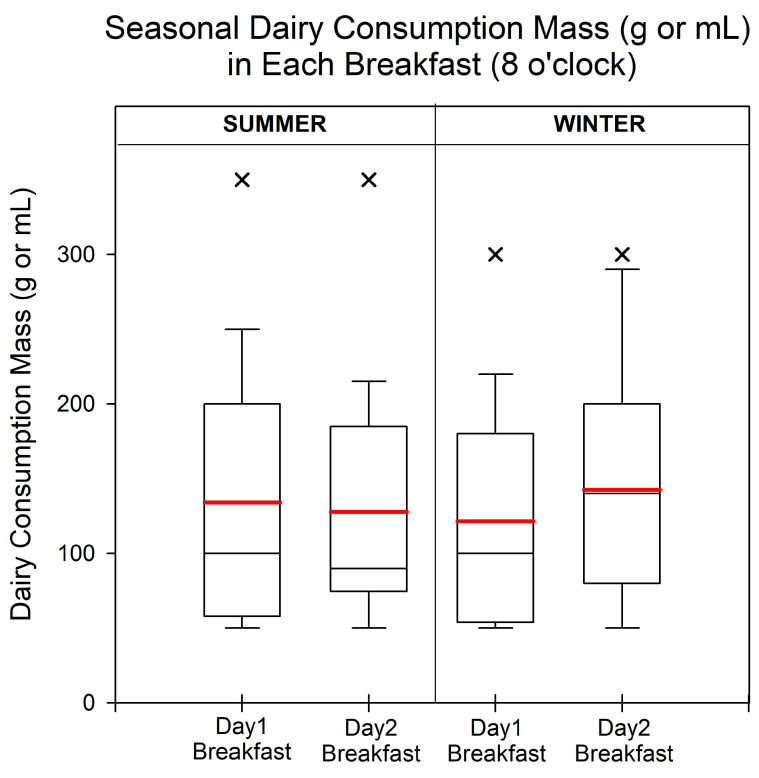
The dairy volume (g or mL) in each meal during summer and winter. Peak dairy product consumption occurred only at 8 o’clock. The red line represents the mean value of the dairy consumption. The box ranges from the 25th to 75th percentiles, and the ends of the whiskers show minimum and maximum data values, excluding outliers. The black and red lines represent the median and mean values of intake, respectively. Cross (×) denotes outliers that are more than 1.5 times the interquartile range.

In Figure 2, the peak consumption of dairy products in both summer and winter occurred between 0800 and 0859. Although we observed dairy consumption at different time periods, the data were insufficient to make a comparison. Detailed information on dairy product consumption is provided in Table S5.

The maximum, median, and minimum consumption levels of dairy products during breakfast were 250 g, 100 g, and 50 g or mL, respectively, on the first day of summer. On the second day, the maximum and median consumption decreased to 220 g and 90 g or mL, while the minimum consumption remained the same, at 50 g or mL. In the winter over the two days, the minimum consumption remained the same, at 50 g or mL, and the maximum consumption increased by approximately 26.1% from 230 to 290 g or mL. Also, the median consumption on the second day (140 g or mL) was slightly higher than that on the first day (100 g or mL). In addition, the participants consumed slightly more dairy products on the first day during summer, whereas the trend was reversed in winter.

### 3.4. Water Consumption

Water-based drinks consumption, such as water, coffee, and tea, exhibited similar patterns throughout both summer and winter (Table S6). In summer, the total cumulative daily water intake ranged from 4398 to 4899 mL. During the morning hours (07:00 to 11:59), approximately 28.8% and 28.3% of the total water consumption occurred. The period between lunch and dinner (12:00 to 17:59) accounted for approximately 38.5% and 37.6% of the total water intake, while the evening hours (18:00 to 23:59) constituted 32.7% and 34.1% of the total intake. In winter, the total daily water intake ranged from 3838 to 4346 mL. Similar to summer, the morning hours accounted for approximately 30.7% and 29.5% of the total water consumption. The period between lunch and dinner represented approximately 40.9% and 40.0% of the total water intake, while the evening hours accounted for 28.4% and 30.5% of the total intake.

**Figure 3:**
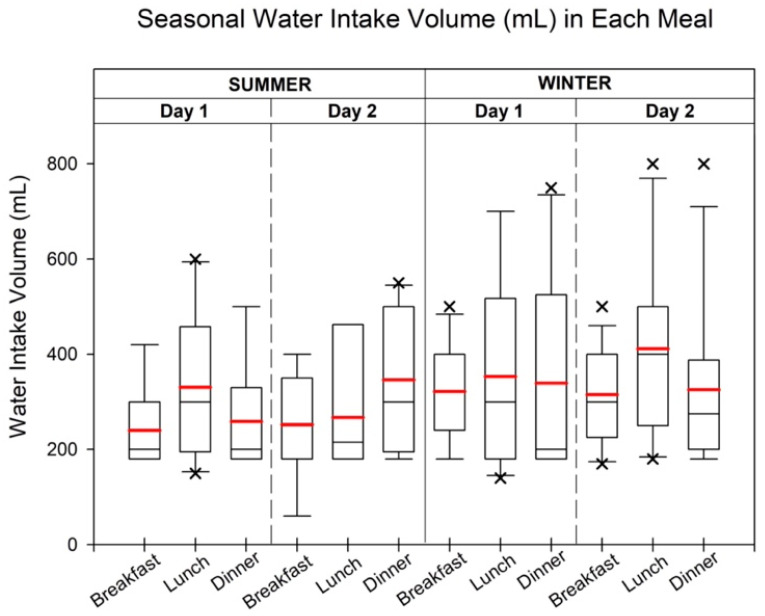
The water intake volume (mL) in each meal during summer and winter. Each water intake time was followed by the highest meat consumption in each meal during the summer and winter. The boxplot extends from the 25th to 75th percentiles, with whiskers marking the minimum and maximum values, excluding outliers. Cross (×) highlights outliers, defined as values exceeding 1.5 times the interquartile range. Median and mean intakes are indicated by black and red lines, respectively.

Figure 3 presents the maximum, minimum, median, and mean hourly water consumption for each day. In summer, the mean water intake during breakfast, lunch, and dinner on the first day was 241.1 mL, 367.8 mL, and 268.0 mL, respectively. On the second day, the mean water intake slightly increased during dinner compared with the first day, with values of 252.2 mL, 305.7 mL, and 384.4 mL for breakfast, lunch, and dinner, respectively. In winter, the mean volume of water intake during each meal was generally higher than in summer. Specifically, the mean intake during breakfast, lunch, and dinner on the first day was 321.8 mL, 353.6 mL, and 339.0 mL, respectively. On the following day, the mean intake for breakfast, lunch, and dinner was 315.4 mL, 453.0 mL, and 325.8 mL, respectively. Additional details regarding the maximum, minimum, and median consumption volumes can be found in Table S6.

### 3.5. Antibiotic Detection and Quantification from the Meat Samples

Triplicates of 34 food and drink samples were tested to determine the presence of the target antibiotics. All foods mentioned in the survey responses from the omnivorous and unknown dietary intake groups in the online survey were included. Most of the detections were over the LOD and LOQ with relatively high accuracy.

Table 2 shows that the MRL is the maximum amount of antibiotic residue that is expected to legally remain in food products. ADI is then calculated based on chronic intake of MRL and a theoretical daily food basket (consisting of 300 g meat, 1500 mL milk, and 100 g eggs). Lastly, TMDI is calculated based on the high quartile bounds of food intake, 65% to 80%, to stress the worst-case scenario or conservative limits. Highly consumed antibiotics in Table 2 have been detected in all environmental samples, including foods and drinking water, over the world.

In Table 3, the concentration of detected antibiotic residues in animal food product samples was calculated based on the survey and chemical analysis results. Non-detected products (organic salted butter, organic unsalted butter, medium cheddar, dairy spray cream, Greek style yoghurt, sweetened probiotic milk, London tap water, and two different water brands) were not included. We observed nine of our target antibiotics except erythromycin in the samples. Interestingly, processed products such as salami, tuna chunks, ham, meatballs, and sausages exceeded the concentration of antibiotics compared to the MRLs. The most exceeded concentration in meat was ENR in sausages (5497.3 μg/kg), which was 141.0 times greater than the MRL (39.0 μg/kg). In addition, the concentration of AMOX in skimmed milk (1481.6 μg/kg) exceeded the MRL by 370.4 times (4 μg/kg).

In beef, eight different products were analysed, including ribeye, sirloin, rump, diced beef, minced beef, corned beef, burger patty, and meatballs. AMOX, AMP, ENR, and TMP were commonly detected in all beef products, whereas AMOX was not detected in meatballs. All detected concentrations were greater than the MRLs. Ten different dairy products were tested, but no target antibiotics were detected in six of the products, including organic salted butter, organic unsalted butter, medium cheddar, dairy spray cream, Greek-style yoghurt, and sweetened yoghurt drink. However, β-lactams and TMP were commonly found in the remaining four products (whole milk, semi-skimmed milk, organic semi-skimmed milk, and skimmed milk). AMP in whole milk was detected below the MRL, but AMOX concentration was 29.6 times higher than the MRL detected in skimmed milk.

We had the highest variety of food products from pork, and all the products contained TMP except sausages. Although TMP was the most frequently detected antibiotic, lipophilic antibiotics such as AMOX and ENR were detected at concentrations exceeding the MRLs. For instance, 137.3 and 108.2 times higher AMOX and ENR than their MRLs were measured in salami, and 141.0 times higher concentration of ENR was detected in sausage. The concentrations of OTC in ham, ENR in unsmoked back bacon, and SDZ in ribs and salty canned pork were lower than their respective MRLs.

In chicken, 11 different products, consisting of five organic and six conventional products, were tested. In general, AMOX, SDZ, and TMP were detected in the products. All OTC and TET detections were lower than the MRLs. CIP concentrations from the chicken breast were also lower than the MRL. ENR in BBQ chicken wings (5492.3 μg/kg) was the highest concentration detected from all samples and exceeded the MRL by 153.2 times. Interestingly, four out of five organic products (drumstick, chicken breast, eggs, and whole chicken) had higher concentrations of antibiotics than the same types of conventional products. Organic drumstick, chicken breast, and egg had 17.0, 609.9, and 14.4% higher concentrations of AMOX compared with conventional products, respectively. In addition, a 31.0% higher concentration of SDZ was detected in organic drumstick. A total of 1404.6 μg/kg of AMOX was detected in organic whole chicken, while AMOX was not detected in the conventional product.

Five different farmed and wild fish products were tested, and OTC, TET, and ENR were commonly detected in wild fish, including mackerel, cod, and haddocks, while β-lactam and sulfonamides were measured in farmed fish, such as salmon and tuna. All the measured concentrations were above the MRLs. ENR was measured at 13.8 times higher than the MRL from haddock fillets and AMOX was detected at a concentration 59.4 times higher than its MRL.

### 3.6. Estimated Meal Intake (EMI) of Antibiotics from Each Meal

Estimation of antibiotic residues consumed via each meal provides valuable insights into the potential exposure of individuals to these antimicrobial agents. In this study, the estimated daily intake formula modified from the US FDA was utilized to calculate the antibiotic intake from each meal. The formula took into account factors such as portion size, the concentration of antibiotics in the consumed foods, and the frequency of consumption. Moreover, the total volume of gut juice in the stomach and duodenum was set to 60 mL on the basis of monitoring the human digestive system using magnetic resonance imaging quantification [27]. By applying these parameters, the study aimed to estimate the antibiotics that individuals may be exposed to during specific meals.

The results showed varying levels of estimated antibiotic residues in different meals. As shown in Table 4, during the first day, the estimated antibiotic intake from breakfast included 141.3 mg/L of amoxicillin and 64.2 mg/L of enrofloxacin, while lunch had an estimated intake of 399.4 mg/L of amoxicillin. Notably, dinner on the first day was estimated to be below the ADI concentration for all antibiotics analysed. Similarly, the estimated antibiotic residues from each meal on the second day varied, with breakfast, lunch, and dinner containing different antibiotic concentrations.

## 4. Discussion

We showed that our survey results are in line with previously published research and national surveys in the UK [26]. In general, meat consumption followed the peaks of water consumption. In both seasons, only pork was consumed during breakfast over the two days. Similarly, chicken and beef were mostly consumed at lunch. Fish and chicken were the most frequently consumed animal products in both seasons. Most participants had meals at regular times without special occasions such as celebrations or irregularly skipping meals. UK adults in the national survey (*n* = 8174) reported consuming pork products such as ham, bacon, and sausages the most at breakfast [28]. Moreover, the participants had the highest consumption of beef > chicken > fish over the rest of the day. It was also determined from the national survey that the most consumed meats in the UK were (in order of highest to lowest) beef, chicken, and fish [28]. It is reasonable to assume that the participants’ dairy products intake is mostly via drinking milk at breakfast. Water intake is directly related to food consumption.

The meat consumption patterns observed in this study exhibit cultural influences, personal preferences, and seasonal variations. The preference for specific types of meat in each meal aligns with the findings of the impact of cultural values and beliefs on meat consumption [29]. For example, the consistent consumption of pork for breakfast reflects cultural norms, whereas the higher average consumption of chicken compared with beef may be influenced by perceptions of chicken as a lean and healthy choice [30]. In addition, seasonal availability and individual taste preferences contribute to variations in meat consumption. Ueland et al. (2022) found that individuals consume more poultry during winter months when other meat sources may be limited, supporting the higher chicken and beef consumption observed during winter lunches [31]. Spence et al. (2021) also emphasized the role of flavour preferences and seasonal associations, explaining the consistent patterns observed between summer and winter meat consumption [32].

These findings have implications for public health initiatives aimed at promoting healthier and sustainable meat consumption. By considering cultural influences, nutritional profiles, and seasonal variations, tailored interventions can be developed. Understanding the complex interplay among individual preferences, cultural norms, and health considerations is crucial. Further research should explore these factors in depth to develop evidence-based strategies. Overall, this study contributes to the growing body of knowledge on meat consumption patterns and informs efforts to promote balanced and sustainable dietary choices.

The analysis of water consumption patterns among university faculties revealed consistent trends in both summer and winter, indicating stable hydration practices regardless of the season [33,34]. These observations align with the findings of previous research on water consumption patterns [35,36]. The consistent water intake during these specific time intervals suggests that individuals prioritize hydration during the morning and lunch hours, possibly to support gut digestion and overall wellbeing [37]. Adequate water intake during these periods can aid in diluting the concentrations of antibiotics ingested through food, thereby potentially reducing their impact on the gut microbiome [38,39]. In addition to the consistent trends in water consumption, the mean intake volumes during breakfast, lunch, and dinner varied between summer and winter. Higher mean intake volumes in winter may be attributed to increased thermoregulatory demands during colder months, leading to higher fluid intake [40,41]. This observation is in line with studies that have shown increased water needs in response to environmental factors [42]. Understanding these variations in water intake throughout the year can inform public health strategies aimed at promoting optimal hydration and managing antibiotic exposure in animal products [27].

The vast majority of those studies reported concentrations that were typically in the ng/L, ng/kg to μg/L, or μg/kg range [43,44,45,46,47,48,49]. Although the WHO has limited the concentration of penicillin to below 100 μg/kg in animal products, greater concentrations have been detected in foods and drinking water [43,44,50,51,52]. The concentrations of amoxicillin and ampicillin in milk were in the range of 28.4 to 96.8 μg/L and in meat were 58.2 to 157 μg/kg [44]. Moreover, concentrations between 0 to 17.8 μg/L of amoxicillin and ampicillin were found in drinking water [43]. Tetracycline and oxytetracycline were detected between 57.0 to 137 μg/L in milk and 82.0 to 691 μg/kg in meat [45]. In drinking water and tap water, 0.09 to 21.1 μg/L of tetracycline and oxytetracycline were detected [47]. Long-term consumption of a trace level of tetracyclines needs to be focused on because of its poor biodegradability, which may accumulate in the body to make a reservoir of pathogens have greater resistance. Samples of poultry meats in Europe demonstrated contamination of sulfadiazine and trimethoprim in ranges of 0.64 to 243 μg/kg [43]. Furthermore, 0.20 to 15.2 μg/L of sulfonamides in drinking water have been reported worldwide [48].

There are no relevant data on erythromycin and tylosin antibiotic pollution in foods and drinking water because their usage has decreased significantly compared with the past decades. However, they have huge potential to become high-risk antibiotics as food consumption and production are projected to increase significantly in South American, Asian, and African countries in the future [53]. Moreover, the FAO designated the concentration of ciprofloxacin and enrofloxacin limit to 2 μg/kg, but edible trout still contained 170 to 1006 μg/kg of enrofloxacin in European countries [46]. Concentrations of up to 6.5 mg/L of ciprofloxacin have been found in drinking water in India [49].

Cattle are routinely given antibiotics to treat and prevent mastitis, which is an infection of the udder and is very common [54]. It can be subclinical, where they show no obvious symptoms, or clinical, which causes painful swelling in one or more quarters of the udder. Mastitis is commonly treated with antibiotics administered as an intramammary directly into the cow’s teat [55]. There are various types of mastitis-causing organisms, including staphylococci. Antibiotics used to treat *Staphylococcus aureus* mastitis include AMOX, AMP, ERY, TYL, ENR, and TMP, which is highly probable with regard to our antibiotic detection from beef and dairy products in Table 3 [56]. Furthermore, mastitis infection can be monitored in herds through cell counts in milk, and farmers are financially penalized by dairy companies for high cell counts [57]. Milk from infected cows must be withheld from sale for the required withdrawal periods. However, fermented or intensively processed products such as yoghurt, cheese, cream, and butter were prevented from using antibiotics, and extra care was taken with antibiotic detections for the higher rate of fermentation or cost-effectiveness [58]. This explains why the milk had antibiotic residues but no detections were observed in fermenting-based products.

The poultry industry is split into two parts: the broiler industry, which produces birds slaughtered at 6–7 weeks of age for the table, and the egg-producing sector, where layers are reared and placed in battery cages at 16–18 weeks for one egg-laying cycle and then killed. Treatment is required for any outbreak of necrotic enteritis, *Colispticaemia salmonellosis* causing mortality, mycoplasma infection, or necrotic dermatitis (*Staphylococcus aureus*) [59]. The antibiotics used for salmonella and *E. coli* may include ENR and AMOX, which are reflected in our results in Table 3. We can assume that CIP is metabolized from ENR, and it is important to regulate the intensive use of ENR because of the incidence of isolates of multidrug-resistant *Salmonella typhimurium DT104* from humans that are resistant to CIP [60].

Swine that are reared indoors receive intensive antibiotic treatment throughout their life until slaughter, usually at under 6 months of age. Most conventional herds are watered or fed with growth promoters during the early stages of growth. It is true that we did not detect any growth promotors from pork products (Table 3), but there are various growth promoter antibiotics used in pig farming that are more cost-effective than our targeted macrolides. For instance, avilamycin, carbadox, flavomycin, olaquindox, spiramycin, and salinomycin [61]. Our detection could be explained by the most conventional herds in which antibiotic treatment starts soon after birth. Piglets are typically treated with AMP, ENR, TMP, and SDZ for *E. coli* enteritis and respiratory disease [62] and slaughtered after six months.

Fish farming products are still contaminated with antibiotics with relatively high concentrations, although the official usage of antibiotics has been significantly reduced from the past due to increased regulation, vaccination, and the segregation of farmed fish by age [63]. Recent studies have raised concerns that antibiotics enter fish farms not only through direct medication, but also through feeding with chicken faeces, which are treated with intensive treatments [64,65]. In Table 3, it is not unreasonable to postulate that AMOX, SDZ, and TMP concentrations from chicken were similar to those from farmed fishes such as salmon and tuna. Moreover, a large number of feed pellets were found in the gut contents of wild fishes such as mackerel, cod, and haddocks near a fish farm in Scotland [66]. Furthermore, wild fishes are more vulnerable to antibiotic aquatic pollution, which is rarely taken into consideration [67]. It is worth noting that most of the antibiotics used are persistent in the environment and spread from farms to surrounding areas where accumulation in sediments may occur [68]. Residues of antibiotic concentrations may far exceed levels accepted for human consumption [66]. In addition, fishes were treated with antibiotics after being caught from the ocean to avoid the pathogenic penalties of regulation [69].

The application of veterinary antibiotics to food-producing animals has led to residues occurring in food products such as beef, chicken, pork, and dairy products, which increases the risks to human health. In addition, the removal of antibiotics from drinking water is highly variable depending on treatment technologies, including activated carbon adsorption, ozonation, membrane filtration, and advanced oxidation processes (AOPs) [70]. According to Liu et al. (2015), the removal of antibiotics was effective using a combination of activated carbon adsorption and ozonation in the water treatment process [71]. Sand biofiltration is expected to be a widely demanded technology because of its low cost [72]. AOPs such as Fenton oxidation and photocatalytic oxidation have demonstrated high efficiencies of antibiotic removal (>90%); however, the formation of various antibiotic by-products is the main concern of AOPs [73]. The properties of antibiotics, including pharmacokinetic characteristics, physicochemical or biological processes, and improper usages, are considered factors influencing the occurrence of antibiotic residues in foods and drinking water [1].

Most hygiene guidelines state that foods should be kept above boiling point for sufficient time to kill harmful pathogens [74]. Although the majority of pathogens are killed in the cooking process, studies have found that the concentration of antibiotic residues in foods is not significantly degraded after cooking at a temperature above 100 °C for more than 30 min [75]. Different cooking practices, including boiling, frying, and grilling, at different cooking times have been examined to understand antibiotic concentration reduction in foods [76,77,78]. Firstly, tetracyclines, including oxytetracycline, tetracycline, chlortetracycline, and doxycycline, were tested to determine the reduction in antibiotic concentrations with different cooking procedures (boiling, microwave, and roasting) at different time ranges (0, 10, 15, 20, 30, 40, 60, and 80 min [76]. A significant reduction in chlortetracycline and doxycycline concentrations was observed with all cooking procedures starting at 30 mins. However, oxytetracycline and tetracycline levels were not reduced by more than 50% at the maximum exposure time of 80 min. Moreover, the concentrations of chlortetracycline and oxytetracycline were reduced by 27.6% and 35.6%, respectively, after boiling milk for 30 mins [78]. Also, 11.1% of antibiotics were inactivated by heating for 30 mins in water [78].

Furthermore, degrading antibiotics, including ciprofloxacin, tylosin, oxytetracycline, and sulfonamides, in beef, chicken, and rabbit meat samples were ineffective in reducing the concentration in the meat samples by boiling and roasting processes [79,80]. For instance, ciprofloxacin was reduced by 17.0% and 22.4% after roasting and boiling chicken muscle, respectively, for 30 min [80]. Furusawa and Hanabusa (2002) tested the degradation effect of boiling, roasting, and microwaving on sulfonamides in chicken muscle [81]. Sulfadiazine appeared to be stable in boiling, roasting, and microwaving methods, with a maximum reduction of 32.3% compared with other sulfonamides, including sulfamethoxazole, sulfamonomethoxine, and sulfaquinoxaline (45.0–61.0%). Interestingly, degradation of tylosin in chicken meatballs under microwaving had the lowest reduction (2.8%) compared with the other antibiotics, while microwaving showed a strong antibiotic reduction [82]. Meanwhile, the concentration of enrofloxacin in raw meat samples was increased by 44.0–310% during grilling and roasting due to the loss of moisture content in the samples [83].

The estimation of antibiotic residues consumed via each meal provides valuable information on the potential exposure of individuals to these antimicrobial agents. The results of this study demonstrate variations in estimated antibiotic residues in different meals, emphasizing the importance of monitoring and minimizing antibiotic residues in food to mitigate the risks associated with antibiotic resistance.

*Lactobacillus* sp., *Escherichia coli*, and *Enterococcus* spp. are the most predominant species in the human duodenum [84]. Considering the human gut microbiota’s susceptibility to antibiotics, it is notable that different bacterial species exhibit varying MICs. According to the EUCAST MIC dataset, *Lactobacillus* sp. displayed MIC levels of up to 32 mg/L for ampicillin. *E. coli* exhibited the highest MIC levels for amoxicillin and ampicillin, reaching up to 512 mg/L and 8 mg/L for enrofloxacin. Meanwhile, *Enterococcus* spp. reported MIC levels of up to 256 mg/L for amoxicillin and ampicillin. Although enrofloxacin MIC data for *Enterococcus* spp. Are unavailable, ciprofloxacin, the main metabolite of enrofloxacin, showed a maximum MIC level of 512 mg/L. In addition to MIC levels, the duration of antibiotic exposure is also critical. For example, to eradicate *E. coli*, 1–3 h of antibiotic exposure is typically required, whereas the bacteria reproduce approximately every 20 min [85]. With a duodenal transition time of approximately 18 mins, which is a third of the minimum time required to eliminate *E. coli*, it is likely that these bacteria would survive chronic exposure to subtherapeutic antibiotic levels.

Although the estimated luminal antibiotic exposure from dietary sources in our study may not reach levels sufficient to eradicate gut microbiota, understanding the potential consequences of chronic luminal exposure is crucial to assessing risks associated with the development of antimicrobial resistance. Future investigations should delve into the intricate interplay between chronic luminal antibiotic exposure and gut microbial communities to provide comprehensive insights into the possible implications of antimicrobial resistance development and overall gut health.

## 5. Conclusions

In conclusion, this study underscores the potential risks of dietary antibiotic exposure, even at subtherapeutic levels, in contributing to the development of antibiotic resistance. The comparison of estimated luminal antibiotic concentrations from meals with prescribed dosages highlights substantial differences, raising concerns about the efficacy of dietary antibiotics. Additionally, the analysis of minimum inhibition concentrations for key gut bacteria emphasizes the complexity of microbial responses. Although dietary exposure may not achieve eradication levels, chronic exposure to subtherapeutic concentrations could foster antimicrobial resistance. This underscores the urgency for stringent regulation of antibiotic residues in food and a deeper investigation into the long-term impacts of chronic luminal antibiotic exposure on gut microbiota and antimicrobial resistance development.

## Figures and Tables

**Table 1:** Profile of participants in the summer and winter dietary intake survey (2021/2022).

**Season (Year)**	**Participant Age**					
	**20s** **(18–29)**	**30s** **(30–39)**	**40s** **(40–49)**	**50s** **(50–59)**	**60s** **(60–69)**	**70s** **(>70)**
	***n***	***n***	***n***	***n***	***n***	***n***
Summer (2021) ^a^	39	13	8	0	1	0
Winter (2022) ^b^	33	17	15	11	3	1
Total (*n* = 141; 21/22)	62	30	23	11	4	1
**Season (Year)**	**Types of Diet**					
	**Omnivorous**	**Vegetarian**	**Vegan**	**Protein-based**	**Halal**	**Unknown**
	***n***	***n***	***n***	***n***	***n***	***n***
Summer (2021) ^a^	30	3	2	1	0	15
Winter (2022) ^b^	70	6	0	0	2	2
Total (*n* = 141; 21/22)	100	9	2	1	2	17

^a^ Summer data were collected from 28 May 2021 to 30 July 2021. ^b^ Winter data were collected from 12 January 2022 to 17 March 2022.

**Table 2:** Theoretical maximum daily intake (TMDI), acceptable daily intake (ADI), and maximum residual level (MRL) of the target antibiotics from the Joint FAO/WHO Expert Committee on Food Additives (JECFA), and limit of detection (LOD) and limit of quantification (LOQ) for each antibiotic. R^2^ for all antibiotics ranged from 0.9993 to 0.9999. Not available data are represented as NA.

Antibiotics	TMDI (μg/person/day; JECFA)	ADI (μg/kg/day; JECFA)	MRL (μg/kg; JECFA)		LOD (μg/L)	LOQ (μg/L)
			Beef, Chicken, Pork, and Fish	Dairy		
Amoxicillin (AMOX)	31.0	2.00	50.0	4.00	10.3	31.3
Ampicillin (AMP)	31.0	2.00	50.0	NA	11.0	33.4
Oxytetracycline (OTC)	370	30.0	200	100	8.50	25.8
Tetracycline (TC)	370	30.0	200	NA	10.9	33.2
Ciprofloxacin (CIP)	NA	2.00	39.0	NA	8.93	27.1
Enrofloxacin (ENR)	NA	2.00	39.0	NA	11.7	35.5
Sulfodiazine (SDZ)	87.5	50.0	100	NA	8.32	25.2
Trimethoprim (TMP)	NA	4.20	50.0	50.0	12.5	38.0
Erythromycin (ENR)	2.75 × 10^4^	700	100	NA	5.75	17.4
Tylosin (TYL)	230	30.0	100	NA	10.0	30.4

**Table 3:** The detected antibiotics (μg/kg or μg/L) in meat and dairy products from different supermarket chains in London.

Type	Name	Concentration of Antibiotics (μg/kg or μg/L)									
		TET	OTC	TMP	SDZ	CIP	ENR	AMOX	AMP	TYL	ERY
Beef	Ribeye *	-	-	90.10	-	-	616.8	674.4	1187	-	-
Beef	Corned beef	-	-	113.7	-	-	62.79	1941	271.5	-	-
Beef	Meatballs	-	-	173.6	-	-	2021	-	348.6	-	-
Beef	Sirloin *	-	-	88.90	-	-	675.2	646.5	659.7	-	-
Beef	Burger patty *	-	-	221.0	-	-	1446	310.9	708.4	-	-
Beef	Rump *	-	-	111.2	-	-	451.8	775.7	988.8	-	-
Beef	Diced beef *	-	-	78.57	-	-	300.9	484.5	538.0	-	-
Beef	Minced beef *	-	-	264.9	-	-	170.3	1612	632.8	-	-
Chicken	Drumsticks *	-	116.0	111.3	654.0	-	-	1199	-	-	-
Chicken	Thighs *	-	-	197.8	1349	-	-	1535	-	-	-
Chicken	Whole chicken	-	-	336.2	3743	151.4	-	-	-	-	-
Chicken	Organic whole chicken	-	-	114.1	987.0	56.78	-	1405	-	-	-
Chicken	Organic drumsticks	-	96.27	96.87	856.6	-	-	1403	-	-	-
Chicken	Organic thighs	-	-	55.23	1029	-	-	1140	-	-	-
Chicken	Chicken wings	-	-	67.52	674.6	-	5976	589.5	-	-	-
Chicken	Free-range eggs	-	-	-	-	-	-	715.6	-	-	-
Chicken	Organic free-range eggs	-	-	-	-	-	-	818.9	-	-	-
Chicken	Organic chicken breast fillets	-	-	75.71	20.00	-	-	233.0	-	-	-
Chicken	Chicken breast fillets *	171.6	-	214.8	53.19	321.3	-	1421	-	327.8	-
Dairy	Whole milk	-	-	95.92	-	-	-	-	36.61	-	-
Dairy	Semi-skimmed milk *	-	-	171.3	-	-	-	760.0	-	-	-
Dairy	Organic semi-skimmed milk	-	-	96.40	-	-	-	-	-	-	-
Dairy	Skimmed milk	-	-	288.8	-	-	-	481.6	-	-	-
Fish	Mackerel fillets	391.0	374.5	-	-	-	-	-	-	-	-
Fish	Salmon fillets	-	-	191.2	-	-	-	-	415.8	-	-
Fish	Tuna Chunks in sunflower oil	-	-	76.39	765.3	-	-	2968	-	-	-
Fish	Cod fillets	648.3	1299	-	-	-	205.4	-	-	-	-
Fish	Haddock fillets	279.3	220.6	-	-	-	538.6	-	-	-	-
Pork	Salami slices *	-	-	81.25	-	425.1	4221	6867	-	-	-
Pork	Pork sausages *	-	-	-	-	-	5497	216.7	-	-	-
Pork	Salty canned pork	-	-	184.6	77.91	-	-	-	-	-	-
Pork	Smoked streaky bacon rashers	-	-	222.2	-	-	-	-	-	-	-
Pork	Unsmoked streaky bacon rashers	-	-	120.0	-	-	-	-	-	-	-
Pork	British pork ribs	-	-	170.5	34.80	-	-	-	-	-	-
Pork	British pork chops	-	-	461.7	116.2	-	-	1616	-	-	-
Pork	British pork belly slices	-	-	107.6	-	-	-	-	-	-	-
Pork	British pork loin	-	-	185.4	1118	-	-	-	-	-	-
Pork	Smoked back bacon rashers	-	-	123.1	-	-	61.68	-	-	-	-
Pork	Unsmoked back bacon rashers	-	-	157.0	-	-	37.24	-	-	-	-
Pork	Ham slices *	-	71.83	305.1	-	-	-	2388	-	-	-

* Products that exceed ADI level of amoxicillin, ampicillin, and enrofloxacin and were selected for the EMI calculation based on the diet survey.

**Table 4:** The average of estimated antibiotic intake per meal (mg/L) during the summer and winter using the EMI equation based on the survey results.

Day	Meals	Estimated Antibiotic Intake per Meal (mg/L)		
		Amoxicillin	Ampicillin	Enrofloxacin
1st	Breakfast	141.3	-	64.20
	Lunch	399.4	-	-
	Dinner	<ADI ^a^	<ADI	-
2nd	Breakfast	132.5	<ADI	80.30
	Lunch	170.7	193.5	194.4
	Dinner	408.1	-	-

^a^ Acceptable daily intake.

## Data Availability

The original contributions presented in the study are included in the Supplementary Materials; further inquiries can be directed to the corresponding author.

## Supplementary Materials

The following supporting information can be downloaded at: https://www.mdpi.com/article/10.3390/toxics12030174/s1, Figure S1: The 10 target antibiotics (tetracycline, oxytetracycline, amoxicillin, ampicillin, sulfadiazine, trimethoprim, erythromycin, tylosin, ciprofloxacin, and enrofloxacin): reconstructed ion chromatogram for [M+H]^+^, *m*/*z* ≈ 445, 461, 366, 350, 251, 291, 734, 916, 332, and 360, respectively; Table S1: Linearity, limit of detection (LOD), limit of quantification (LOQ), and R^2^ of each antibiotic’s calibration curve; Table S2: The LTPE method validation using LC–MS analysis. Calibration curves and LTPE methods by parameters accuracy (%) and relative standard deviation (RSD; %) using triplicates of nominal concentration (50, 100, and 500 μg/L) and measured concentration (μg/L); Table S3: The recovery of LTPE method using 100 µg/L of 10 antibiotic mixture stock solution, and the recovery of using triplicates of pork chop matrix spiked with 100 μg/L of 10 antibiotics mixture; Table S4: Minimum, maximum, median, and mean consumption (g) of meat products; Table S5: Minimum, maximum, and mean consumption (g or mL) of dairy products; Table S6: Minimum, maximum, median, and mean intake (mL) of water.
