# Supplementary material for: Antibiotic Residues in UK Foods: Exploring the Exposure Pathways and Associated Health Risks
Source: Toxics. 2024 Feb 24;12(3):174. doi: 10.3390/toxics12030174 (PMC10975662; doi:10.3390/toxics12030174)
Supplement: Supplementary file 1 [file toxics-12-00174-s001.zip › toxics-2860175-SI.pdf]

Supplementary Materials

Antibiotic Residues in UK Foods: Exploring the Exposure Pathways and Associated Health Risks

Jegak Seo [1,*], Frank Kloprogge [2], Andrew M. Smith [3], Kersti Karu [4] and Lena Ciric [1]

[1] Healthy Infrastructure Research Group, Department of Civil, Environmental and Geomatic Engineering, University College London, Gower Street, London WC1E 6BT, UK
[2] Institute for Global Health, University College London, Rowland Hill Street, London NW3 2PF, UK
[3] Eastman Dental Institute, University College London, Rowland Hill Street, London NW3 2PF, UK
[4] Department of Chemistry, University College London, 20 Gordon Street, London WC1H 0AJ, UK
* Correspondence: jegak.seo.20@ucl.ac.uk

Keywords: diet survey; food contamination; antibiotic residues; low-temperature partitioning extraction; exposure modelling

Table S1. Linearity, limit of detection (LOD), limit of quantification (LOQ) and, R^2 of each antibiotic's calibration curve.

Classes	Antibiotics	Linearity	LOD (μg/L)	LOQ (μg/L)	R^2
Tetracyclines	Tetracycline	0.9995	10.949	33.179	0.9995
	Oxytetracycline	0.9997	8.498	25.750	0.9997
Penicillin	Amoxicillin	0.9995	10.345	31.347	0.9995
	Ampicillin	0.9995	11.018	33.387	0.9995
Sulfonamides	Sulfadiazine	0.9997	8.324	25.224	0.9997
	Trimethoprim	0.9993	12.534	37.980	0.9994
Macrolides	Erythromycin	0.9999	5.751	17.428	0.9999
	Tylosin	0.9996	10.027	30.385	0.9996
Quinolones	Ciprofloxacin	0.9996	8.933	27.071	0.9996
	Enrofloxacin	0.9994	11.707	35.475	0.9994

Figure S1. The 10 target antibiotics (tetracycline, oxytetracycline, amoxicillin, ampicillin, sulfadiazine, trimethoprim, erythromycin, tylosin, ciprofloxacin, and enrofloxacin): reconstructed ion chromatogram for $[M+H]^+$, *m/z* ≈ 445, 461, 366, 350, 251, 291, 734, 916, 332, and 360, respectively.

Tetracycline

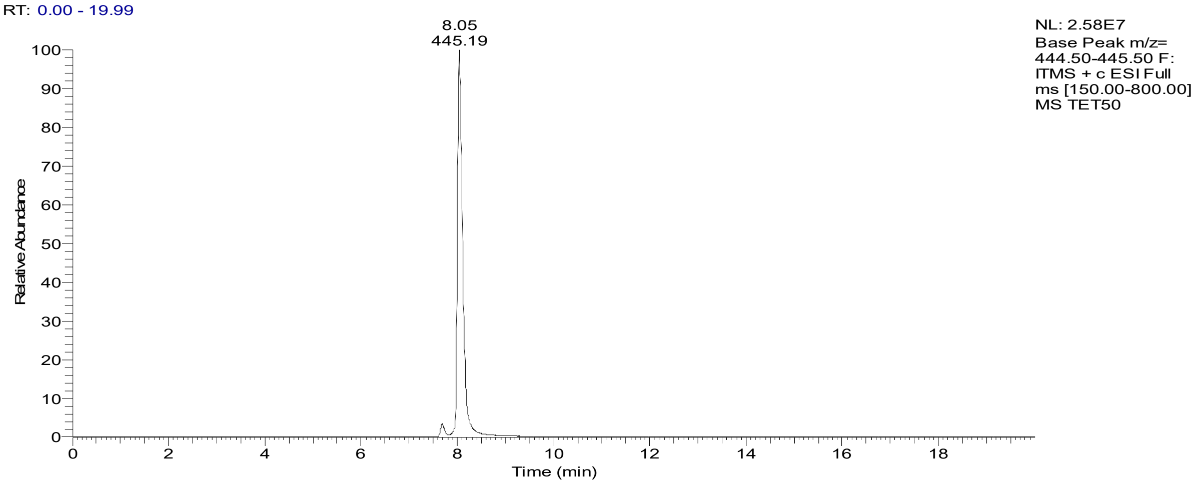

Oxytetracycline

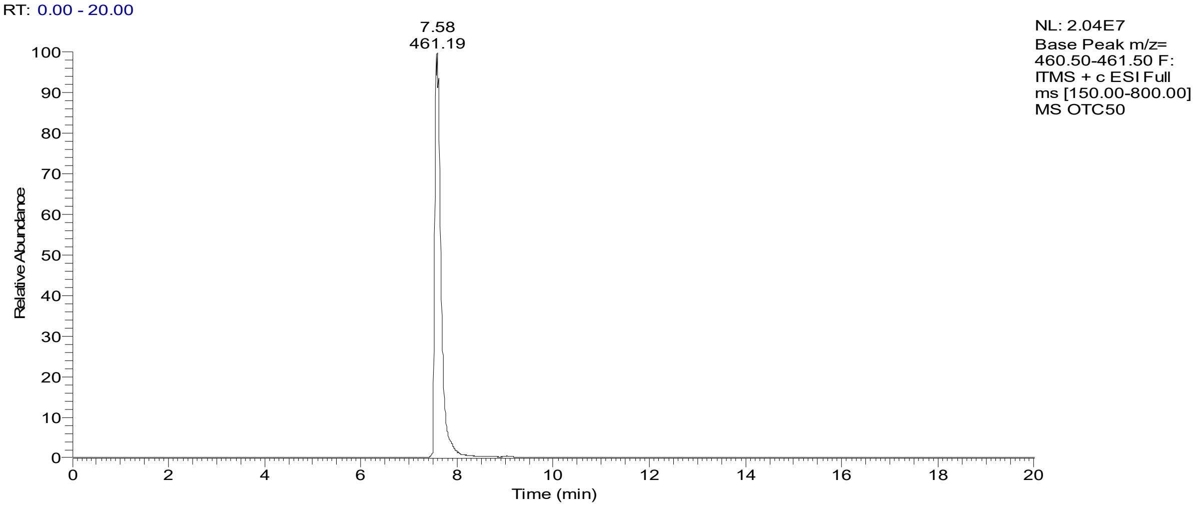

Amoxicillin

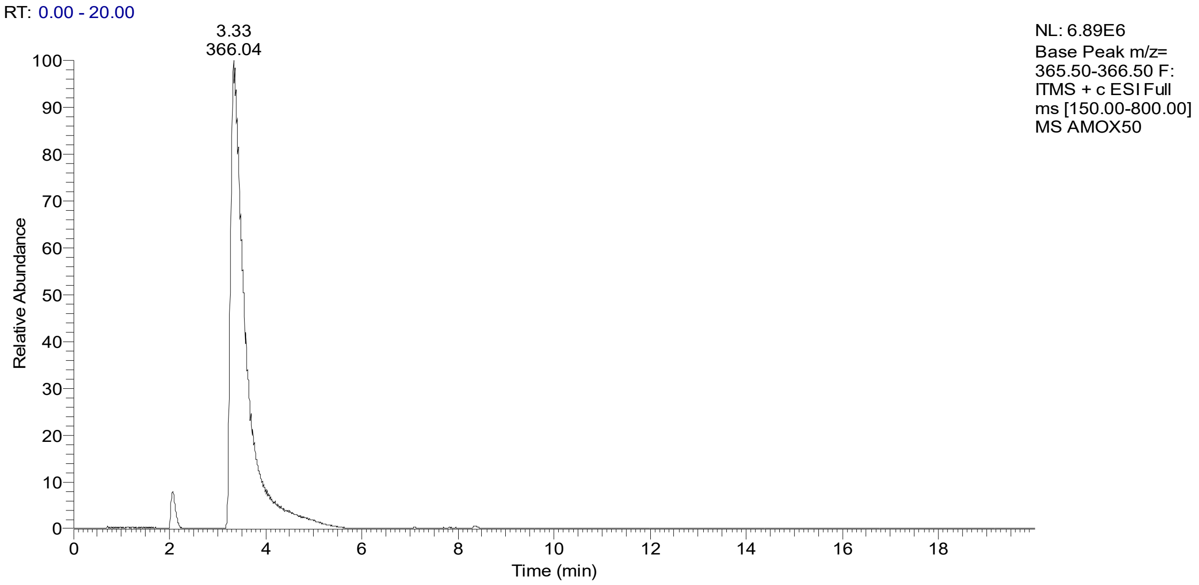

Ampicillin

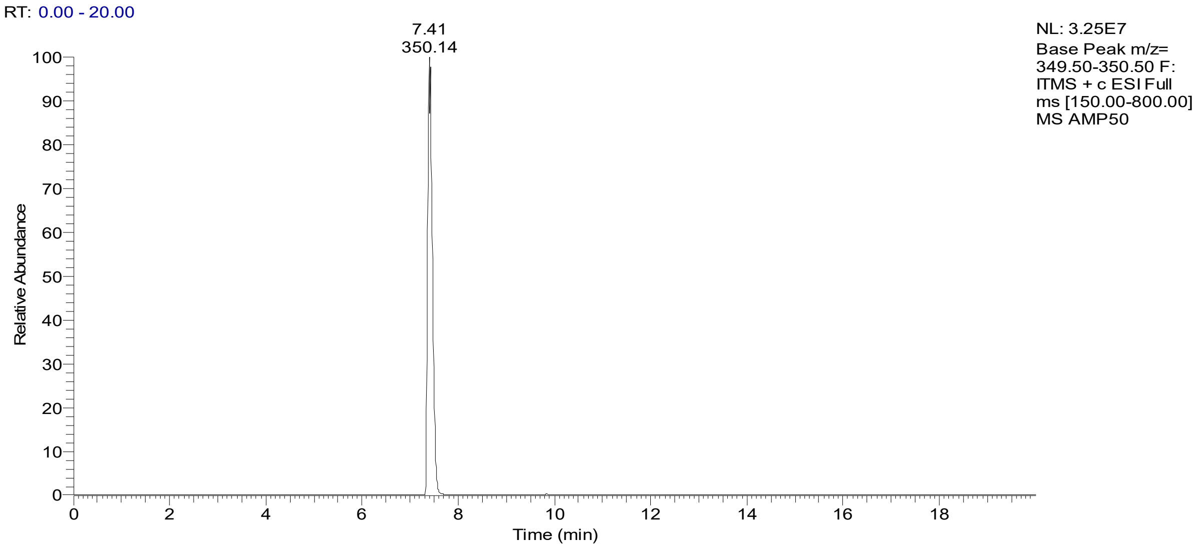

Sulfadiazine

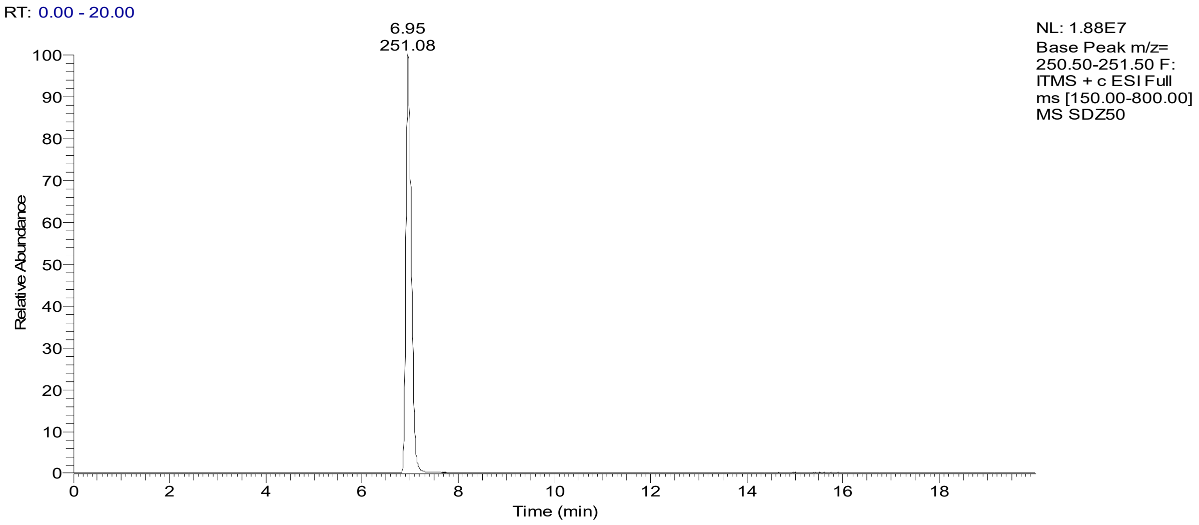

Trimethoprim

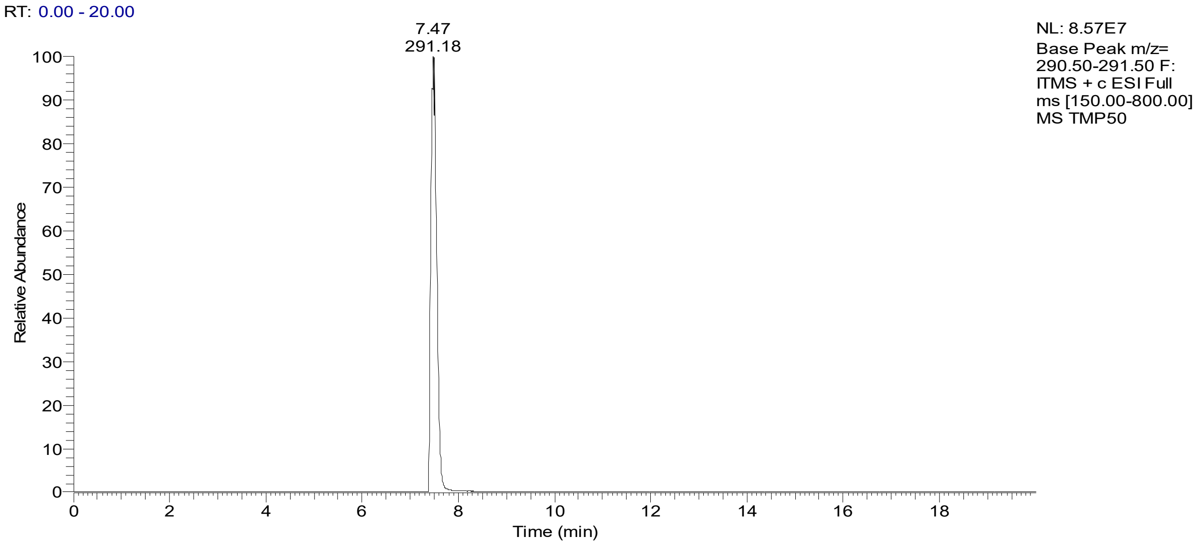

Erythromycin

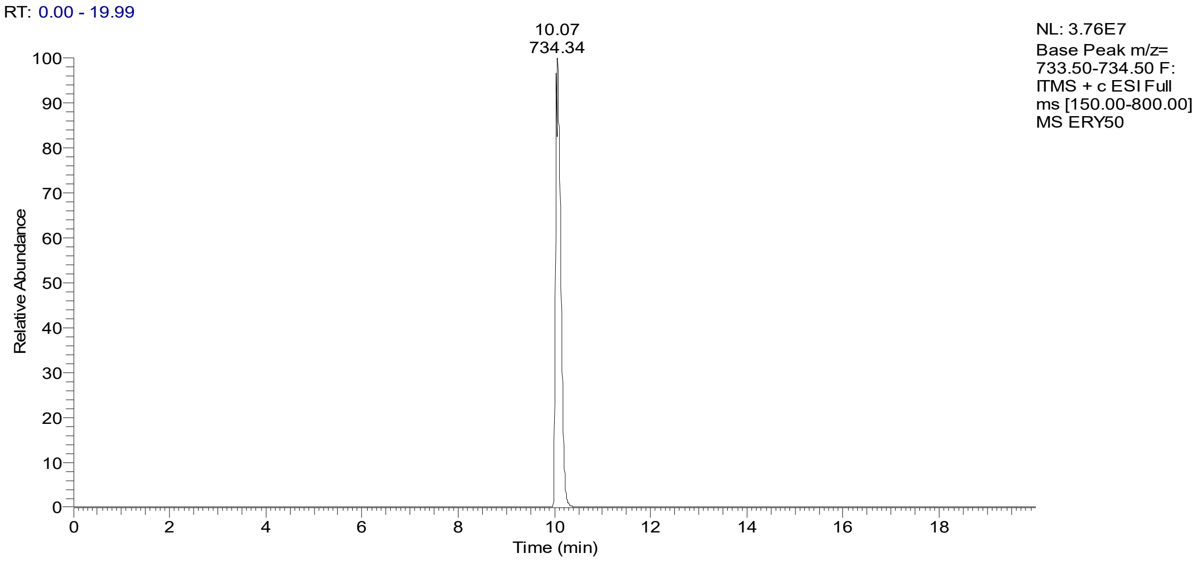

Tylosin

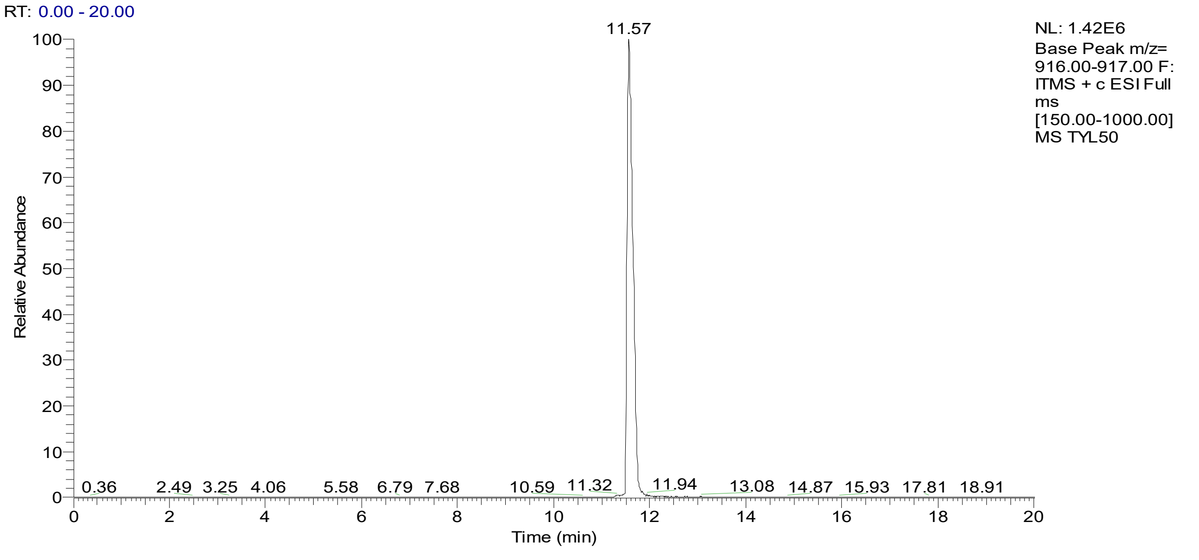

Ciprofloxacin

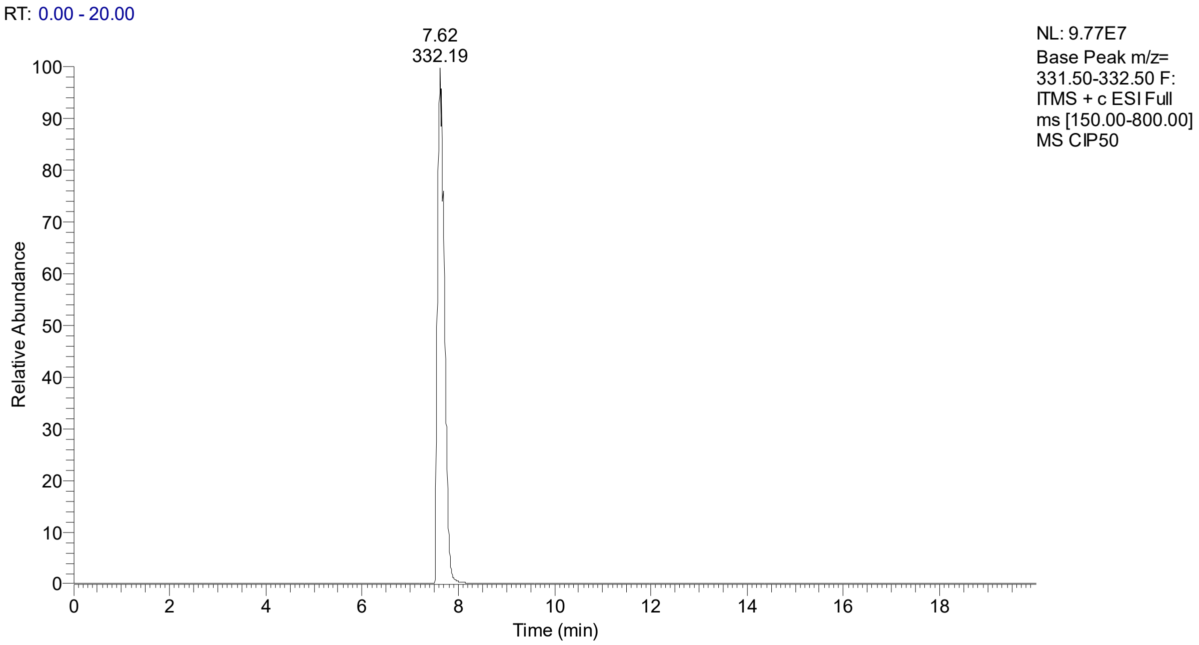

Enrofloxacin

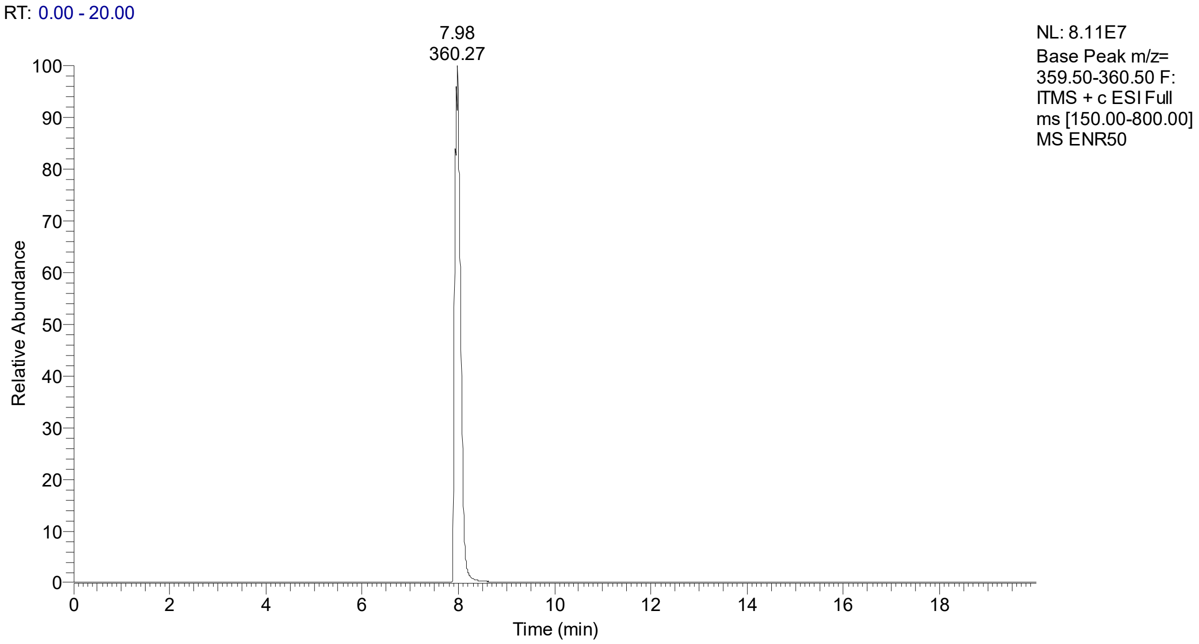

Table S2. The LTPE method validation using LC-MS analysis. Calibration curves and LTPE methods by parameters accuracy (%) and Relative Standard Deviation (RSD; %) using triplicates of nominal concentration (50, 100, and 500 µg/L) and measured concentration (µg/L).

Mean AB Standards Validation							
**Antibiotic**	**MW**	**RT**	**Measured Area**	**Nominal concentration (µg/L)**	**Measured concentration (µg/L)**	**Accuracy (%)**	**RSD (%)**
Tetracycline	445	8.13	195195	50.00	48.97	97.94	0.32
Oxytetracycline	461	7.73	166300	50.00	54.13	108.25	0.34
Amoxicillin	366	3.51	69293	50.00	52.72	105.44	0.51
Ampicillin	350	7.41	241966	50.00	55.61	111.22	0.20
Sulfadiazine	251	6.97	129273	50.00	48.67	97.34	0.44
Trimethoprim	291	7.53	846428	50.00	51.41	102.82	0.11
Erythromycin	734	10.04	226506	50.00	52.19	104.38	0.87
Tylosin	916	11.53	9916	50.00	50.54	101.08	0.37
Ciprofloxacin	332	7.76	418218	50.00	48.60	97.19	0.39
Enrofloxacin	360	8.06	704085	50.00	52.79	105.58	0.10

Mean AB Standards Validation							
**Antibiotic**	**MW**	**RT**	**Measured Area**	**Nominal concentration (µg/L)**	**Measured concentration (µg/L)**	**Accuracy (%)**	**RSD (%)**
Tetracycline	445	8.06	389463	100.00	101.92	101.92	0.04
Oxytetracycline	461	7.72	332537	100.00	104.75	104.75	0.08
Amoxicillin	366	3.52	138540	100.00	105.32	105.32	0.21
Ampicillin	350	7.46	482493	100.00	106.51	106.51	0.06
Sulfadiazine	251	6.91	257510	100.00	101.13	101.13	0.13
Trimethoprim	291	7.50	1693450	100.00	103.23	103.23	0.01
Erythromycin	734	10.04	445852	100.00	104.10	104.10	0.03
Tylosin	916	11.63	19256	100.00	102.15	102.15	0.76
Ciprofloxacin	332	7.76	841564	100.00	101.33	101.33	0.02
Enrofloxacin	360	8.05	1408338	100.00	102.26	102.26	0.02

Mean AB Standards Validation							
**Antibiotic**	**MW**	**RT**	**Measured Area**	**Nominal concentration (µg/L)**	**Measured concentration (µg/L)**	**Accuracy (%)**	**RSD (%)**
Tetracycline	445	8.07	1923085	500.00	519.42	103.88	0.60
Oxytetracycline	461	7.64	1660534	500.00	509.00	101.80	0.34
Amoxicillin	366	3.62	690274	500.00	522.92	104.58	0.48
Ampicillin	350	7.45	2419374	500.00	515.28	103.06	0.51
Sulfadiazine	251	6.92	1289961	500.00	523.26	104.65	0.42
Trimethoprim	291	7.52	8476638	500.00	518.45	103.69	0.12
Erythromycin	734	10.01	2221920	500.00	524.26	104.85	0.28
Tylosin	916	11.68	91891	500.00	506.37	101.27	0.92
Ciprofloxacin	332	7.83	4182029	500.00	517.20	103.44	0.43
Enrofloxacin	360	8.08	7036687	500.00	497.68	99.54	0.03

Mean AB LTPE Validation							
**Antibiotic**	**MW**	**RT**	**Measured Area**	**Nominal concentration (µg/L)**	**Measured concentration (µg/L)**	**Accuracy (%)**	**RSD (%)**
Tetracycline	445	7.97	340633	100.00	88.61	88.61	0.31
Oxytetracycline	461	7.65	295535	100.00	93.48	93.48	0.03
Amoxicillin	366	1.94	117015	100.00	88.97	88.97	0.68
Ampicillin	350	7.39	414491	100.00	92.12	92.12	0.76
Sulfadiazine	251	6.91	236253	100.00	92.44	92.44	0.77
Trimethoprim	291	7.43	1515047	100.00	92.32	92.32	0.63
Erythromycin	734	10.05	397927	100.00	92.76	92.76	0.75
Tylosin	772	11.55	17439	100.00	92.12	92.12	0.46
Ciprofloxacin	332	7.68	772549	100.00	92.73	92.73	0.60
Enrofloxacin	360	7.97	1199189	100.00	87.57	87.57	0.18

Table S3. The recovery of LTPE method using 100 µg/L of 10 antibiotic mixture stock solution, and the recovery of using triplicates of pork chop matrix spiked with a 100 µg/L of 10 antibiotics mixture.

**Mean Spiked Meat Validation**							
**Antibiotic**	**RT**	**Measured Area**	**AB standard nominal concentration (µg/L)**	**Total AB concentration from spiked meat (µg/kg)**	**AB concentration from non-spiked meat (µg/kg)**	**Accuracy of spiked meat (%)**	**Accuracy of AB LTPE (%)**
Tetracycline	8.06	345155	100.00	89.84		89.84	88.61
Oxytetracycline	7.62	303293	100.00	95.84		95.84	93.48
Amoxicillin	2.05	2249514	100.00	1708.64	1616.09	92.55	88.97
Ampicillin	7.48	414539	100.00	92.13		92.13	92.12
Sulfadiazine	6.81	523468	100.00	209.94	116.19	93.75	92.44
Trimethoprim	7.68	9084446	100.00	555.45	461.68	93.77	92.32
Erythromycin	10.37	402559	100.00	93.85		93.85	92.76
Tylosin	11.58	17858	100.00	94.43		94.43	92.12
Ciprofloxacin	7.87	785378	100.00	94.33		94.33	92.73
Enrofloxacin	8.06	1228007	100.00	89.60		89.60	87.57

Table S4: Minimum, maximum, median and, mean consumption (g) on meat products.

Summer (g)									
Meat Type (Time)	Day 1				Meat Type (Time)	Day 2			
	Max.	Min.	Median	Mean		Max.	Min.	Median	Mean
Pork (0800-0859)	100	40	80	88	Pork (0800-0859)	90	40	80	86
Chicken (1300-1359)	600	100	200	236	Beef (1300-1359)	300	100	200	194
Fish (1800-1859)	400	100	180	198	Chicken (1900-1959)	400	100	200	222
**Winter (g)**									
Meat Type (Time)	Day 1				Meat Type (Time)	Day 2			
	Max.	Min.	Median	Mean		Max.	Min.	Median	Mean
Pork (0800-0859)	110	60	80	81	Pork (0800-0859)	100	60	80	77
Chicken (1300-1359)	450	100	250	248	Beef (1300-1359)	300	100	200	196
Fish (1900-1959)	300	100	190	195	Chicken (1900-1959)	450	100	275	269

Table S5. Minimum, maximum and mean consumption (g or mL) on dairy products.

Time	Summer (g or mL)						Winter (g or mL)					
	Day 1			Day 2			Day 1			Day 2		
	Min.	Max.	Mean	Min.	Max.	Mean	Min.	Max.	Mean	Min.	Max.	Mean
0600-0659	-	-	-	-	-	-	-	-	-	-	-	-
0700-0759	50	250	88	50	50	50	50	250	88	50	50	50
0800-0859	50	250	130	50	220	131	50	230	122	50	290	142
0900-0959	150	150	150	-	-	-	150	150	150	-	-	-
1000-1059	-	-	-	100	100	100	-	-	-	100	100	100
1100-1159	-	-	-	-	-	-	-	-	-	-	-	-
1200-1259	-	-	-	-	-	-	-	-	-	-	-	-
1300-1359	50	108	69	50	50	50	50	100	67	50	100	67
1400-1459	-	-	-	50	50	50	100	180	-	50	100	75
1500-1559	-	-	-	-	-	-	180	180	-	-	-	-
1600-1659	100	100	100	100	100	100	100	100	100	100	100	100
1700-1759	-	-	-	100	100	100	-	-	-	100	100	100
1800-1859	65	65	65	65	65	65	65	180	123	65	65	65
1900-1959	-	-	-	50	50	50	-	-	-	50	50	50
2000-2059	180	180	180	-	-	-	180	180	180	40	300	-
2100-2159	-	-	-	-	-	-	-	-	-	-	-	-
2200-2259	-	-	-	-	-	-	-	-	-	-	-	-
2300-2359	-	-	-	-	-	-	-	-	-	-	-	-

Table S6. Minimum, maximum, median, and mean intake (g or mL) of water.

Time	Summer (mL)								Winter (mL)							
	Day 1				Day 2				Day 1				Day 2			
	**Min.**	**Max.**	**Median**	**Mean**	**Min.**	**Max.**	**Median**	**Mean**	**Min.**	**Max.**	**Median**	**Mean**	**Min.**	**Max.**	**Median**	**Mean**
0700-0759	350	350	350	350	200	350	275	275	120	350	150	207	200	300	200	233
0800-0859	180	420	200	241	60	400	250	252	180	500	320	322	170	500	300	315
0900-0959	60	700	225	278	40	500	325	282	150	500	225	268	180	500	250	304
1000-1059	60	350	200	202	60	350	225	210	40	350	120	140	140	350	215	219
1100-1159	180	250	200	210	60	180	150	130	80	250	140	140	60	300	150	158
1200-1259	120	500	180	243	180	500	250	306	40	400	135	164	180	600	325	408
1300-1359	150	1140	250	368	180	850	300	374	140	700	300	354	180	1000	375	453
1400-1459	150	700	350	376	120	500	200	250	100	400	200	219	120	300	180	201
1500-1559	150	500	250	303	60	350	180	242	50	400	150	166	180	500	300	310
1600-1659	180	750	465	465	60	500	180	247	100	400	200	244	60	200	100	126
1700-1759	40	500	300	285	180	350	275	270	100	400	300	275	180	350	300	276
1800-1859	180	500	225	268	180	300	250	254	80	500	190	213	100	500	250	254
1900-1959	-	1050	360	398	180	1050	250	384	180	750	200	339	180	800	275	326
2000-2059	150	700	190	263	180	500	200	288	100	500	190	260	100	360	200	203
2100-2159	180	360	200	235	150	360	200	228	180	200	200	193	100	300	180	194
2200-2259	180	250	215	215	180	180	180	180	120	180	180	160	180	200	190	190
2300-2359	200	200	200	200	180	200	250	227	150	200	175	175	170	180	180	177
